# Design of Substrate Stretchability Using Origami-Like Folding Deformation for Flexible Thermoelectric Generator

**DOI:** 10.3390/mi9070315

**Published:** 2018-06-22

**Authors:** Kana Fukuie, Yoshitaka Iwata, Eiji Iwase

**Affiliations:** Department of Applied Mechanics, Waseda University, 3-4-1 Okubo, Shinjuku-ku, Tokyo 169-8555, Japan; fukuie@iwaselab.amech.waseda.ac.jp (K.F.); iwata5116@ruri.waseda.jp (Y.I.)

**Keywords:** stretchability, thermoelectric generator, flexible device, origami

## Abstract

A stretchable thermoelectric (TE) generator was developed by using rigid BiTe-based TE elements and a non-stretchable substrate with origami-like folding deformation. Our stretchable TE generator contains flat sections, on which the rigid TE elements are arranged, and folded sections, which produce and guarantee the stretchability of a device. First, a simple stretchable device with a single pair of p-type and n-type BiTe-based TE elements was designed and fabricated. The TE elements were sandwiched between two folded polyimide-copper substrates. The length of the wiring between the flat sections changed from 1.0 mm in the folded state to 1.8 mm in the deployed state. It was also confirmed that the single-pair device could generate power in both the folded and deployed states. After this, a stretchable TE generator with eight pairs of p-type and n-type BiTe-based TE elements connected in series was created. The stretchable TE generator was capable of withstanding a stretching deformation of 20% and could also produce an output voltage in both the folded and deployed states.

## 1. Introduction

In this paper, a thermoelectric (TE) generator with bendability and stretchability is proposed in order to allow the TE generator to be attached to a non-flat surface. Conventionally, a TE generator that is used for recycling waste heat is composed of rigid TE elements and a ceramic plate. As a result, these conventional TE generators lack flexible properties, such as bendability and stretchability [1,2]. Therefore, its use is limited to a flat heat source. However, a flexible TE generator is required as there is frequently a need to attach generators to a heat source with a non-flat surface, such as piping or the human body. Recently, TE generators with bendability [3,4,5,6,7,8,9,10,11,12] and/or stretchability [13] were developed by using a deformable TE element (e.g., a carbon nanotube (CNT)-polystyrene (PS) composite) or a rubber-based stretchable substrate (e.g., polydimethylsiloxane (PDMS)). However, deformable TE conversion materials based on polymer composites unfortunately have inferior TE conversion efficiency compared to the rigid BiTe-based TE conversion materials, while the stretchable substrates using rubber have a narrow usable temperature range of 213–423 K [14].

Therefore, it is conceivable to realize a stretchable TE generator using a rigid BiTe-based TE conversion material with high TE conversion efficiency and origami-like folding deformation, which consists of a bendable but non-stretchable polymer substrate with a wide usable temperature range. Recently, the kirigami or origami-like structure has been used for stretchable devices [15,16,17,18,19]. Expansion-contraction deformation of the entire device is possible by connecting the rigid TE elements with foldable metal wiring. This is a feasible solution because expansion and contraction by folding does not require the substrate material to have stretchability. Thus, it is possible to select a material by considering its heat resistance and thermal characteristics. In addition, since folding occurs via local bending deformation, a flexible TE generator that is capable of deformation through expansion and contraction as well as bending can be realized by using a rigid TE element and non-stretchable substrate.

## 2. Experimental Section

### 2.1. Fabrication

**Figure 1:**
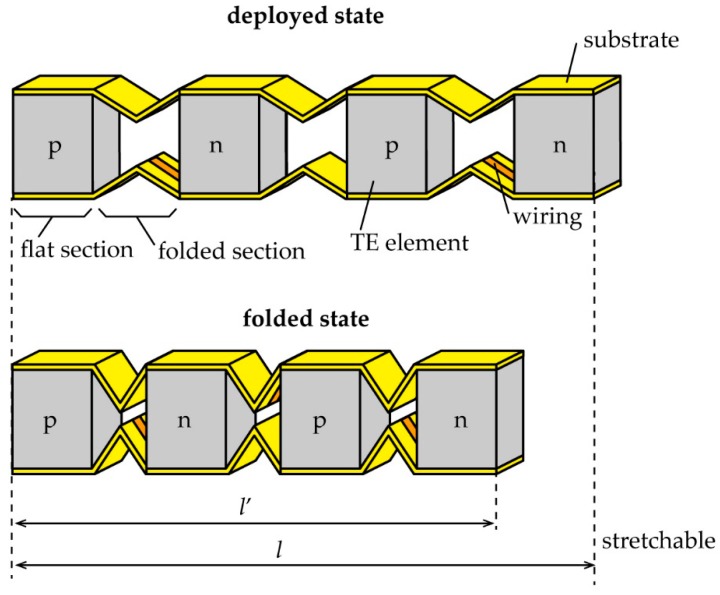
Stretchable substrate using origami-like folding deformation.

The shape of the stretchable TE generator, which is proposed in this research, is shown in Figure 1, which demonstrates that it is capable of folding deformation. A polyimide-copper (Cu) film was used as a bendable but non-stretchable substrate with a wide usable temperature range. It is well known that polyimide has a wide usable temperature range of 4–673 K [20]. The stretchability of our TE generator is realized by connecting the rigid TE elements with the folded wiring parts and by reducing the distance between the TE elements. In this study, p-type and n-type TE elements were alternately arranged and were electrically connected in order to create a π structure.

**Figure 2:**
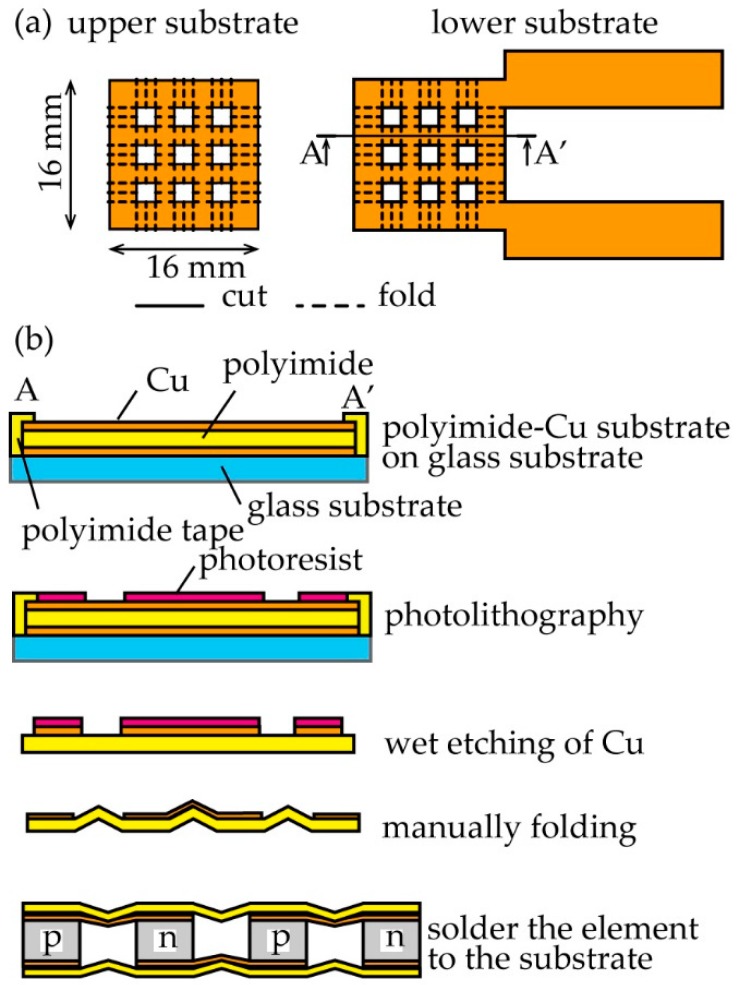
Design and fabrication process of the flexible thermoelectric (TE) generator: (**a**) Cutting and folding lines; and (**b**) fabrication process by photolithography.

The fabrication process is shown in Figure 2. Using a cutting plotter (CE 6000-40, GRAPHTEC, Kanagawa, Japan), a polyimide-Cu substrate (Panasonic Corporation, Osaka, Japan. R-F 786 W; polyimide: 12.5 μm; Cu: 9 μm) was cut as shown in Figure 2a. The lower and upper substrates are shaped as squares of 16 × 16 mm with and without a pad for a lead wire, respectively, with each of these squares having nine 2-mm-long square holes. The cut polyimide-Cu substrate was fixed to a glass substrate using polyimide tape. A photoresist was spin-coated on the Cu layer and patterned to a wire shape. After the Cu layer was wet-etched, the photoresist was removed. Following this, both of the substrates were folded into a shape consisting of mountains and valleys (Figure 2b), thereby fabricating a substrate with stretchability. Finally, TE elements (Toshima Seisakusho, Saitama, Japan; p-type: Bi_0.3_Sb_1.7_Te_3_; n-type: Bi_2_Te_3_) were mounted onto flat sections of the folded substrate using the cream solder (SMX-H05, Sun Hayato Co., Ltd., Tokyo, Japan). The size of each TE element is 2 mm × 2 mm × 1 mm.

### 2.2. Method

First, we aimed to confirm whether thermoelectricity could be generated using the stretchable substrate in its folded state. A pair of p-type and n-type TE elements were arranged in order to have a π-type structure, before a device in which the TE elements were connected by folded wiring was fabricated. The characteristics of the TE generator using folded wiring were evaluated by using a pair of TE generators. The electric power of the TE generator with respect to the temperature produced by the heat source before and after folding the pair of TE elements was measured. The open-circuit voltage *V*_TEG_ (V) of the TE generator is a value determined from the temperature difference that the TE generator is subjected to and the Seebeck coefficient of the device. Therefore, the Seebeck coefficient of the device can be obtained from the open-circuit voltage *V*_TEG_ (V) of the TE generator. Furthermore, the TE generator has an internal resistance *R*_TEG_. Connecting the TE generator to the load causes a voltage drop to occur, whereupon the electric power changes according to the current flowing in the circuit. Therefore, the relationship between the voltage and the electric current with respect to the current and the internal resistance can be determined. The electric power that can be taken out of the TE generator was measured by placing a source meter in the load resistance part. First, a temperature difference was applied between the top and bottom surfaces of the TE generator. This was achieved by placing the TE generator on the ceramic heater and warming the bottom surface. A metal block of 20 mm × 21 mm × 30 mm was placed on the top surface to dissipate heat. The output of the TE generator was connected to a source meter (2614B, Keithley, Instruments, Cleveland, OH, USA) and voltage was applied, before the value of the current at that time was measured. The temperature of the ceramic heater was adjusted from 313 K to 393 K in increments of 20 K. The room temperature at the time of measurement was 297.8 K.

**Figure 3:**
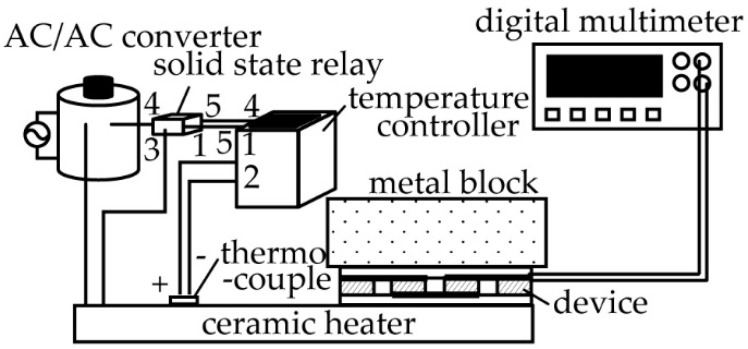
Experimental setup for measuring the output voltage of the flexible TE generator.

It is conceivable that the conditions of contact with the heat source might be different between a single pair and eight pairs of TE generators. Thus, the output voltage of the device with respect to the temperature provided by the heat source to the eight-pair TE generator before and after folding was also measured. A schematic diagram of the measurement setup is shown in Figure 3. A micro-ceramic heater was used to generate heat by supplying a voltage to the heater (MS-2, Sakaguchi E.H Voc Corp., Tokyo, Japan) using an AC/AC converter (Yamabishidenki Co., Ltd., Tokushima, Japan. bolt slider). The temperature of the micro-ceramic heater was measured by attaching a thermocouple to it. Feedback control using the temperature controller (E5CC-RW0AUM-000, Omuron, Kyoto, Japan) was performed to regulate the heat generation temperature of the micro-ceramic heater. Subsequently, the fabricated TE generator was placed on the micro-ceramic heater, while a cylindrical metal block with a diameter of 65 mm and a height of 28 mm was placed on the device in order to allow the top surface of the device to cool down. A digital multimeter (model 2000, Keithley Instruments, Cleveland, OH, USA) was used to measure the output voltage of the device. The temperature of the micro-ceramic heater was increased from 313 K to 393 K in increments of 20 K, with the output voltage of the device being measured during this period of time. The room temperature at the time of measurement was 297.7 K.

## 3. Result and Discussion

**Figure 4:**
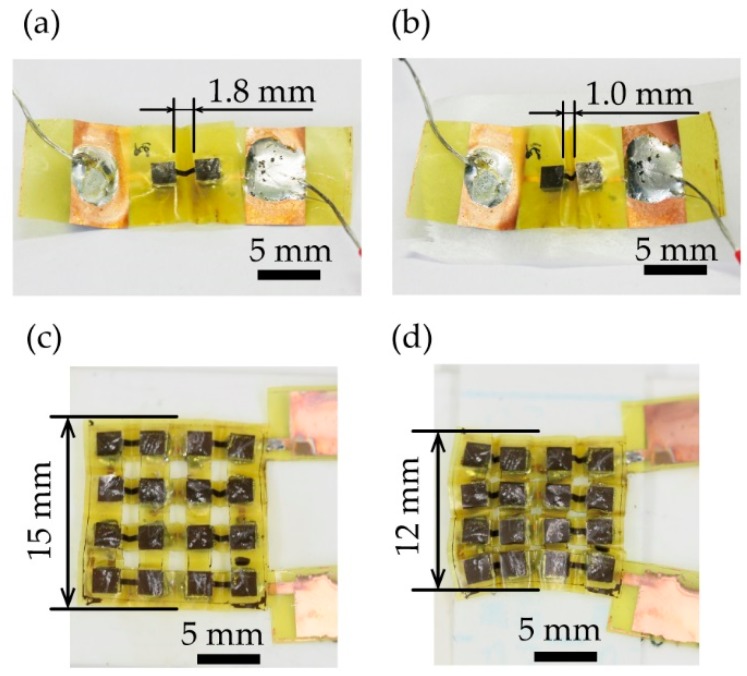
Photographic images of the fabricated devices. A single pair of TE generators in (**a**) the deployed state and (**b**) the folded state. Eight pairs of TE generators in (**c**) the deployed state and (**d**) the folded state.

First, the stretchability of the fabricated devices, which are shown in Figure 4, was evaluated. In the deployed and folded states, the separation between the pair of TE generators was 1.8 mm and 1.0 mm, respectively (Figure 4a,b). Figure 4c shows the deployed device after mounting eight pairs of TE elements. The total length of the device, excluding the padding, was 15 mm. On the other hand, Figure 4d shows the entire device in the folded state with a total length of 12 mm. This result indicates that a TE generator that is capable of a stretching deformation of 20% with respect to the deployed devices was realized.

**Figure 5:**
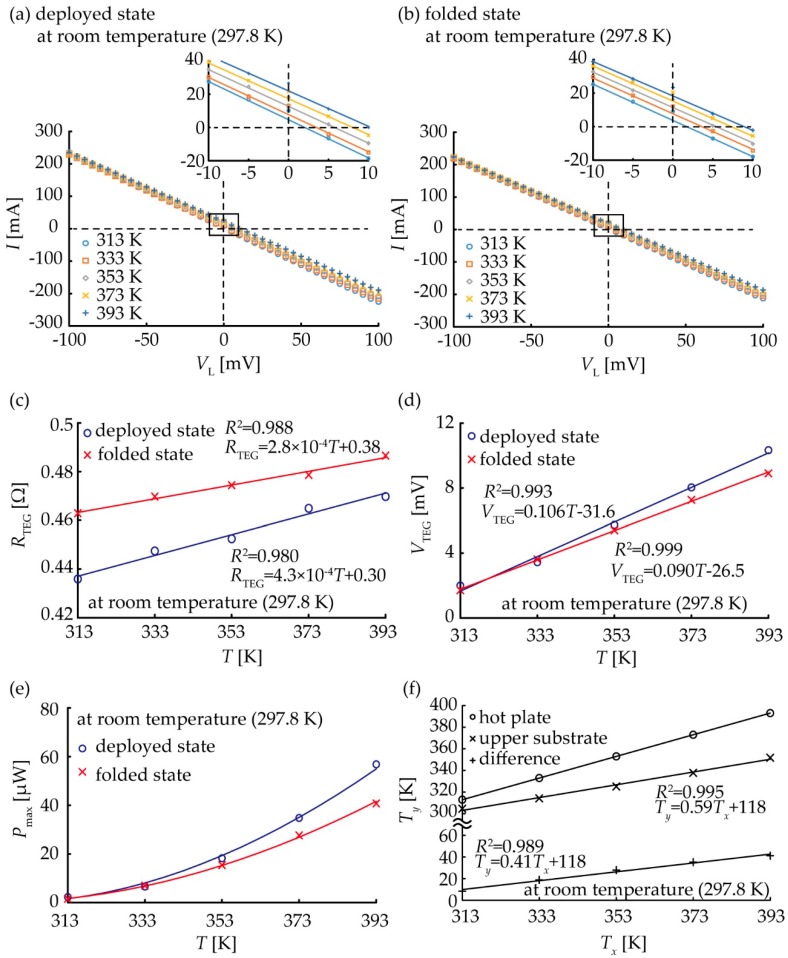
Measurement results of the characteristics of the devices in the case of a single pair of p-type and n-type TE conversion elements: Relationship of the output current to the input voltage of the source meter for the (**a**) deployed and (**b**) folded states; (**c**) Relationship between the internal resistance and the temperature of each of the deployed and folded states; (**d**) Relationship between open circuit voltage and temperature; (**e**) Relationship between maximum output power and temperature; and (**f**) Temperature difference between the top and bottom surfaces of a single pair of p-type and n-type TE elements in the deployed state.

Next, the basic characteristics of the TE generator containing a single pair of TE elements were measured. Figure 5a,b show the relationships between the current value and input voltage of the deployed and folded states, respectively. The TE generator has good linearity in both the deployed and folded states. The relationship between the input voltage and current of the TE generator can be expressed as:

(1) $$V_{L}=-R_{\mathrm{TEG}}I+V_{\mathrm{TEG}}$$

where *V*_TEG_ (V) is the open-circuit voltage of the TE generator, *V*_L_ (V) is the input voltage of the source meter, *R*_TEG_ (Ω) is the internal resistance of the TE generator and *I* (A) is the current measured by the source meter. Therefore, the internal resistance *R*_TEG_ of the TE generator with a single pair of TE elements was calculated from the gradients of the fitting lines in Figure 5a,b. The open-circuit voltage with respect to the temperature can be obtained. Figure 5c shows a plot of the internal resistance of the single pair of TE elements obtained from the slope of Figure 5a,b. Figure 5d plots the open-circuit voltage of the TE generator with the single pair of TE elements with respect to the temperature of the ceramic heater from Figure 5a,b. Since it is an electromotive force, the absolute values were taken and plotted. The output voltage *V*_TEG_ is *V*_L_ when *I* = 0. The fitting lines of the deployed and folded states have the regression coefficients *R*^2^ = 0.993 and *R*^2^ = 0.999, respectively. When the ceramic heater temperature was 393 K, the deployed device received 10.3-mV and the folded device received 8.90-mV open circuit voltage. When the temperature of the ceramic heater increased, a temperature difference was generated between the top and the bottom surfaces of the device, resulting in an increase in the open-circuit voltage. The Seebeck coefficient of the device, which is the regression coefficient of the filling lines in Figure 5d, was 0.106 ± 0.005 mV/K and 0.090 ± 0.001 mV/K for the deployed and folded states, respectively. The Seebeck coefficients measured for both states are close to each other, but statistically different. The reason for the difference in the Seebeck coefficients might be that the contact thermal resistance in the folded device is higher. The relationship between the electric power and the temperature of the ceramic heater is shown in Figure 5e. As the temperature difference increases, the output also increases. When the temperature of the ceramic heater was 393 K, this resulted in an output power of 56.8 μW for the deployed device and 40.7 μW for the folded device. It was possible to generate power with the TE generator using folded wiring. In Figure 5, the device characteristics were evaluated by using the heating temperature, because the contact thermal resistance change should be included in the device characteristics in our experiments. In general, the temperature difference within the TE generator is proportional to the supplied temperature difference [21], which is the temperature difference between the hotplate and room temperature (Figure 5f). The difference between the gradients of the fitting lines in Figure 5c,d originating from the change in the contact thermal resistance was considered. In this experiment, since a metal block was placed on the device, the pressure, which should be the reduced difference in contact thermal resistance, was applied to the device. Therefore, it is conceivable that the thermal contact conditions between the deployed state and folded state differed according to folding deformation.

**Figure 6:**
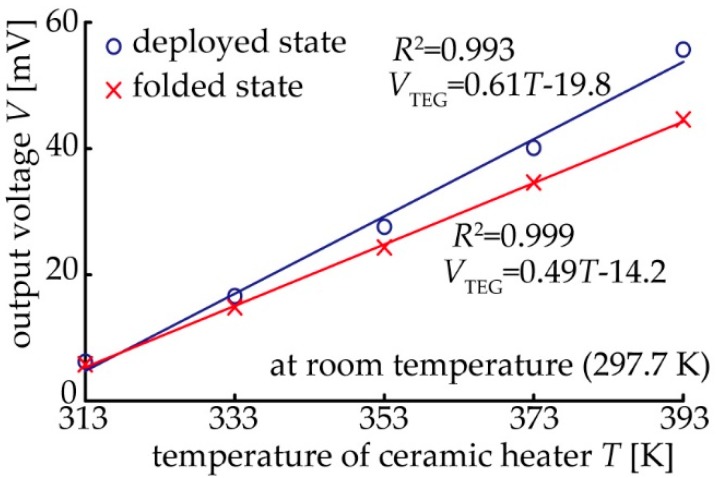
Relationship between the temperature of ceramic heater and output voltage.

The open-circuit voltage was also measured for the TE generator with eight pairs of TE elements. This is because the area, in which the heat source is installed and the contact thermal conductivity is affected, can be varied by using either a single pair or eight pairs of TE elements. The result of the output voltage with respect to the temperature of the ceramic heater is shown in Figure 6. The result confirmed that the fitting line for each device in the deployed and folded states has good linearity. In both cases, the output voltage also tends to increase in proportion to the temperature input to the lower substrate. Consequently, the performance of the device as a TE generator is effective. However, the output voltage of the folded state is lower than that of the deployed device. The Seebeck coefficient of the device was 0.61 ± 0.03 mV/K and 0.49 ± 0.01 mV/K for the deployed and folded states, respectively. The changing ratio of the Seebeck coefficient of the device for the eight-pair device in Figure 6 is larger than that for the single-pair device in Figure 5d. We believe that this is because the contact thermal resistance can easily increase due to the difficulty of maintaining uniform folding in a device with a large area. It is difficult to transfer heat due to the creation of a gap between the substrate and the heater or the metal block. The above results confirm that it is possible to realize a TE generator using a substrate with a folded structure.

## 4. Conclusions

In this study, we realized a stretchable TE generator with a deformation ratio of 20%. The rigid BiTe-based TE elements are connected with folded wiring. A device consisting of a single pair of TE elements was fabricated and the TE characteristics of this device were evaluated. For the single pair of elements, it was confirmed that electric power is generated because of the difference in temperature between the upper and lower surfaces of the device. After this, a TE generator with a larger area was demonstrated by electrically connecting a pair of devices in series using folded wiring. The measurements showed that the output voltage of the TE generator that is capable of expansion and deformation tends to rise in proportion to the input temperature in the case of both the deployed and folded devices. The Seebeck coefficient of the devices was different between the deployed and folded state. This is because the contact thermal resistance becomes high due to folding deformation. In this research, a stretchable TE generator using a non-stretchable substrate was developed. This means that a stretchable TE generator with few constraints can be fabricated from substrate materials. Therefore, since materials with good thermal conductivity could also be used, it is possible to realize a highly efficient flexible TE generator by using our method.
